# From participation to inclusion: Advancing lived experience integration in forensic psychiatric care and research

**DOI:** 10.1192/j.eurpsy.2026.10951

**Published:** 2026-07-31

**Authors:** E. Drewelow, M. Daum, K. Gerullis, I. Kilimann, O. Bienetzky, S. Teipel, B. Völlm

**Affiliations:** 1 Clinic for Forensic Psychiatry; 2 Clinic for Psychosomatic Medicine and Psychotherapy, Rostock University Medical Center; 3 Deutsches Zentrum für Neurodegenerative Erkrankungen, Rostock, Germany

## Abstract

**Introduction:**

Over the past three years, the PART project has established an advisory board for forensic psychiatric patients. The acronym PART derives from the term ‘participation’. While the initial focus was on involving people with lived experience in research, the current phase emphasizes their comprehensive inclusion across all stages of research. This shift marks a move beyond traditional participatory approaches toward a model that recognizes experiential knowledge as an equal component of both research and therapeutic practice.

**Objectives:**

The overarching aim is to create ethical guidelines that maximize adherence to ethical standards in VR therapy within forensic settings, ensuring responsible application that safeguards patients’ rights and well-being.

**Methods:**

We present a case study of a VR project co-developed with members of the PART advisory board, consisting of current and former forensic psychiatric patients. Since spring 2025, board members having already contributed to research question development, method selection and design of an interview guide, and continuing in the coming months with data collection and analysis. Their involvement, as part of developing ethical guidelines for VR therapy in forensic settings, was evaluated through qualitative interviews and surveys assessing experiences, benefits, and challenges.

**Results:**

Preliminary findings show that genuine inclusion of lived experience enhances project relevance, patient engagement, and reveals new care improvement perspectives. Members report empowerment and a stronger sense of agency. Challenges include maintaining meaningful involvement and addressing systemic barriers. The VR project shows how lived experience informs technological and therapeutic interventions. Affected individuals’ involvement increases acceptance of new therapeutic approaches. Through equal and sustained participation, members expand methodological competencies and expertise. This inclusion leads to more targeted research and improved outcomes.

**Conclusions:**

The transition from participatory research to comprehensive lived experience inclusion is a significant advance in forensic psychiatric care and research. Integrating experiential knowledge at all research stages enriches scientific outcomes and clinical practice, opening new horizons for patient-centered mental health care. Co-developing ethical guidelines for VR use in forensic therapy is a key achievement, contributing to responsible innovation and safeguarding patient rights.

**Disclosure of Interest:**

None Declared

